# Effects of dry needling and kinesio taping in temporomandibular dysfunction: a randomized trial

**DOI:** 10.1590/1806-9282.20251053

**Published:** 2025-12-05

**Authors:** Ayse Duran, Elif Esra Ozmen, Bayram Sonmez Unuvar

**Affiliations:** 1 Karamanoğlu Mehmetbey University, Faculty of Medicine, Department of Ear, Nose and Throat Diseases – Karaman, Türkiye.; 2 Karamanoğlu Mehmetbey University, Department of Oral and Maxillofacial Surgery – Karaman, Türkiye.; 3 KTO Karatay University, Faculty of Health Sciences, Department of Audiology – Konya, Türkiye.

**Keywords:** Temporomandibular joint disorders, Myofascial pain, Dry needling, Athletic Tape, Pain, Sleep quality

## Abstract

**INTRODUCTION:**

The aim of the study was to compare the effects of dry needling and kinesio taping on pain intensity, mandibular range of motion, sleep quality, and depression levels in patients diagnosed with myogenous temporomandibular dysfunction (TMD).

**METHODS:**

Ninety patients with TMD were included in this two-center randomized controlled trial. Participants were randomly assigned to three groups: Group A (dry needling), Group B (kinesio taping), and Group C (control—conservative recommendations). Interventions were applied over 6 weeks. Primary outcome measures included the visual analog scale, Beck Depression Inventory, Pittsburgh Sleep Quality Index, and mandibular mobility assessment.

**RESULTS:**

Both intervention groups demonstrated significant improvements in pain reduction, increased mandibular range of motion, and improved sleep quality (p<0.05). The dry needling group showed superior outcomes compared to the other groups. While both the dry needling and kinesio taping groups showed within-group improvements in depression scores, between-group differences were not statistically significant (p=0.464), and lateral mandibular movements did not differ significantly among groups.

**CONCLUSION:**

Dry needling and kinesio taping are effective, safe, and clinically feasible interventions for the management of TMD. Dry needling, in particular, appears more effective in reducing pain and improving function. These findings highlight the importance of incorporating conservative physical therapy approaches within multidisciplinary TMD treatment protocols.

## INTRODUCTION

Temporomandibular dysfunctions (TMDs) represent a group of complex conditions characterized by dysfunction of the temporomandibular joint (TMJ), masticatory muscles, and surrounding supportive structures, with a multifactorial etiology^1^. TMD is recognized as one of the most common causes of orofacial pain, often presenting with muscle tenderness, joint sounds, and limitations in mandibular motion^2^. While the etiology of TMD remains unclear, several contributing factors have been proposed, including parafunctional habits, trauma, occlusal abnormalities, emotional stress, and postural disorders^1,3^.

Epidemiological data reveal significant variability in TMD prevalence across populations, likely influenced by cultural, geographic, and methodological factors^3,4^. Notably, TMD symptoms are more frequently reported in women, potentially due to hormonal influences, pain sensitivity, and psychosocial variables^5,6^.

TMD is associated with chronic pain, sleep disturbances, and psychological distress, all of which can significantly impair quality of life. Therefore, management strategies should aim not only to alleviate symptoms but also to target underlying pathophysiological mechanisms^7,8^. Conservative treatment options are typically the first-line approach and may include physical therapy, therapeutic exercise, behavioral interventions, and complementary modalities^8,9^.

Kinesio taping (KT) is a non-invasive intervention that has gained popularity in TMD treatment. It provides proprioceptive feedback, enhances local circulation, and modulates muscle activity through the application of elastic tapes on the skin^10^. In contrast, dry needling (DN) is an invasive technique that targets myofascial trigger points to reduce pain and relieve muscle spasm. Clinical and experimental studies suggest that DN enhances blood flow, improves oxygenation, and suppresses pain mediators^11,12^.

Accordingly, this study aims to evaluate and compare the effects of DN and KT on pain, depression, sleep quality, and mandibular function in individuals with myogenous TMD. The study hypothesizes that both physical therapy approaches will offer superior clinical outcomes compared to traditional conservative recommendations.

## METHODS

### Study design and ethical approval

This prospective, two-center, randomized controlled trial was conducted between March 1, 2025, and June 1, 2025, at the Faculty of Dentistry and the Department of Otorhinolaryngology, Faculty of Medicine, Karamanoğlu Mehmetbey University University. Ethical approval was obtained from the university's Clinical Research Ethics Committee on [15/01/2025], under the decision number [15-2025/09]. Informed consent was obtained from all participants, who were also informed of their right to withdraw from the study at any stage.

Randomization was performed using a sealed envelope method, with group allocations carried out by an independent researcher unaware of participant identities. The study protocol adhered to the CONSORT 2010 guidelines.

### Participants

The study included volunteer individuals aged 18–60 who presented with symptoms such as jaw pain, earache, and/or joint sounds and were diagnosed with myogenous TMD. The diagnosis was based on the diagnostic criteria for temporomandibular dysfunctions (DC/TMD) developed by Dworkin and LeResche^13^.

Exclusion criteria included intra-articular TMD, skeletal deformities, malignancies, infectious diseases, systemic rheumatologic or hematologic disorders, fibromyalgia, psychiatric diagnoses, major head and neck anatomical pathologies, regular use of analgesic medications, and individuals unable to cooperate. All evaluations and interventions adhered to the principles of the Declaration of Helsinki and were conducted by different blinded assessors.

### Sample size calculation

The required sample size was calculated using G*Power 3.1 software (Heinrich Heine University, Düsseldorf, Germany). Based on an alpha level of 0.05, an effect size of 0.40, and 80% power (1–β), a minimum of 66 participants was needed for analysis of variance (ANOVA) across three groups. To enhance statistical power and account for potential dropouts, 30 participants were included in each group. The final sample size was deemed sufficient to meet the assumptions of parametric testing^14^.

### Outcome measures

#### Sociodemographic assessment

Demographic data, including age, sex, educational background, marital status, and monthly income, were collected to assess baseline comparability across groups^14^.

#### Pain assessment

Pain levels were measured using the visual analog scale (VAS), a 10 cm horizontal line where 0 indicates no pain and 10 represents the most intense pain imaginable. Participants were instructed to mark their pain levels both at rest and during functional activities^14^.

#### Depression assessment

Psychological status was evaluated using the Beck Depression Inventory (BDI), a validated 21-item self-report scale. Each item is rated on a 0–3 scale, with total scores ranging from 0 to 63. Scores of 0–13 indicate no depression, 14–24 suggest mild-to-moderate depression, and ≥25 indicate severe depression^15^.

#### Sleep quality

The Pittsburgh Sleep Quality Index (PSQI) was used to assess subjective sleep quality over the past month. It consists of 19 items across multiple domains, yielding a total score ranging from 0 to 21. A total score of ≥5 indicates clinically significant poor sleep quality. PSQI demonstrates a diagnostic sensitivity of 89.6% and specificity of 86.5%^16^. All assessments were administered by the same trained investigator according to a standardized protocol.

### Interventions

#### Dry needling group

Dry needling was performed using sterile stainless steel needles measuring 13 mm in length and 0.25 mm in diameter, with a plastic cylindrical guide tube. Prior to needle insertion, the skin was disinfected with an antiseptic solution. The needles were inserted into the masseter and temporalis muscles, targeting trigger points located beneath the zygomatic arch and approximately 2.5 cm anterior to the tragus along the mandibular angle. Each session involved a clockwise rotation of the needle for 10 min, followed by a counterclockwise rotation for an additional 10 min. Sessions lasted 20 min and were applied three times per week for a total of 6 weeks^17^.

#### Kinesio taping group

KT was performed using Y-shaped, cotton-based, and latex-free elastic tape applied to the masseter muscle. The tape was worn for 4 days and re-applied weekly over a 6-week period. This method aimed to provide proprioceptive input, support soft tissues, and modulate muscle activity^10^.

#### Control group

Participants in the control group received general conservative recommendations, including the consumption of soft foods, use of analgesics when necessary, bilateral mastication, and application of superficial heat. Participants were asked to document their use of analgesics. Heat application was advised to promote local circulation and reduce muscle spasm^18^. All participants were instructed in a standardized home exercise program^14^.

### Statistical analyses

All statistical analyses were performed using SPSS version 25.0 (IBM Corp., Released 2017, IBM SPSS Statistics for Windows, Version 25.0, Armonk, NY: IBM Corp.). The normality of continuous variables was assessed using the Kolmogorov-Smirnov and Shapiro-Wilk tests. Descriptive statistics were presented as mean±standard deviation (SD) for continuous variables and as frequencies and percentages for categorical variables.

For baseline comparisons among the three groups, one-way ANOVA was used for continuous variables, and the chi-square test was applied for categorical variables. To evaluate within-group and between-group differences before and after the interventions, a two-way repeated-measures ANOVA was conducted. Where appropriate, Bonferroni post hoc tests were used for pairwise comparisons. The significance level was set at p<0.05 for all analyses.

## RESULTS

There were no statistically significant differences among the three groups in terms of demographic and clinical characteristics (p>0.05; Table 1).

**Table 1:** Demographic information and clinical characteristics.

Variables		DN	KT	Control	p
Age (years)		37.6±9.93	35.6±8.26	35.6±4.75	
Gender	Male	14 (46.7%)	11 (36.7%)	16 (53.3%)	0.427
	Female	16 (53.3%)	19 (63.3%)	14 (46.7%)	
Marital status	Married	17 (56.7%)	12 (40%)	14 (46.7%)	0.429
	Single	13 (43.3%)	18 (60%)	16 (53.3%)	
Clicking on mouth opening	Yes	22 (73.3%)	18 (60%)	21 (70%)	0.516
	No	8 (26.7%)	12 (40%)	9 (30%)	
Clicking on mouth closing	Yes	21 (70%)	18 (60%)	21 (70%)	0.638
	No	9 (30%)	12 (40%)	9 (30%)	
Psychiatric history	Yes	17 (56.7%)	13 (43.3%)	18 (60%)	0.392
	No	13 (43.3%)	17 (56.7%)	12 (40%)	
Splint use	Yes	6 (20%)	9 (30%)	9 (30%)	0.600
	No	24 (80%)	21 (70%)	21 (70%)	
Ear pain	Yes	10 (33.3%)	12 (40%)	12 (40%)	0.828
	No	20 (66.6%)	18 (60%)	18 (60%)	
Jaw locking	Yes	19 (63.3%)	18 (60%)	19 (63.3%)	0.954
	No	11 (36.7%)	12 (40%)	11 (36.7%)	
Eating restriction	Yes	13 (43.3%)	16 (53.3%)	12 (40%)	0.559
	No	17 (56.7%)	14 (46.7%)	18 (60%)	
Sleep problem	Yes	14 (46.7%)	15 (50%)	17 (56.7%)	0.733
	No	16 (53.3%)	15 (50%)	13 (43.3%)	
Headache	Yes	12 (40%)	13 (43.3%)	11 (36.7%)	0.870
	No	18 (60%)	17 (56.7%)	19 (63.3%)	

F: one-way analysis of variance in repeated measures; DN: dry needling; KT: kinesio taping. Note: p<0.05. Pain intensity was similar across groups at baseline (p=0.150), but differed significantly post-intervention (p<0.001). Pain decreased significantly in both DN and KT groups (p<0.001), and modestly in the control group (p=0.032). Change scores confirmed a significant treatment effect (p<0.001).

Depression scores showed no group differences at baseline (p=0.551) or after treatment (p=0.960). However, significant within-group improvements were noted in the DN (p=0.011) and KT (p=0.001) groups, but not in controls (p=0.124). Between-group comparisons showed no significant change (p=0.464) (Table 2).

**Table 2:** Comparison of values preintervention and postintervention.

		Groups; means (SD)			TI^a^	TI^b^
		DN	KT	Control	p	p
Pain intensity	Pre	7.20 (0.80)	7.50 (0.63)	7.56 (0.67)	0.150	**<0.001**
	Post	4.33 (0.75)	6.03 (0.96)	7.20 (0.71)	**<0.001**	
	TI^c^	**<0.001**	**<0.001**	**0.032**		
	Cohen'd	**2.844**	**1.326**	**0.412**		
Depression	Pre	20.23 (5.39)	21.23 (5.04)	19.87 (4.77)	0.551	0.464
	Post	18.10 (4.33)	18.03 (4.01)	18.30 (3.54)	0.960	
	TI^c^	**0.011**	**0.001**	0.124		
	Cohen'd	**0.497**	**0.642**	0.289		
Mouth opening	Pre	28.93 (4.38)	31.20 (3.54)	29.67 (3.64)	0.077	**<0.001**
	Post	33.10 (4.89)	31.23 (3.74)	29.60 (3.84)	**0.012**	
	TI^c^	**<0.001**	0.919	0.807		
	Cohen'd	**1.173**	0.018	0.449		
Maximum mouth opening	Pre	32.20 (4.45)	34.07 (3.51)	32.03 (3.99)	0.076	**<0.001**
	Post	35.77 (4.47)	34.37 (3.16)	31.80 (3.33)	**<0.001**	
	TI^c^	**<0.001**	0.423	0.269		
	Cohen'd	**1.561**	0.148	0.205		
Right lateral jaw movement	Pre	6.07 (1.01)	5.97 (0.92)	6.03 (1.03)	0.921	0.155
	Post	6.63 (0.99)	6.63 (0.81)	6.07 (1.28)	0.138	
	TI^c^	**<0.001**	**<0.001**	0.088		
	Cohen'd	**0.730**	**0.879**	0.322		
Center lateral jaw movement	Pre	6.07 (1.01)	5.97 (0.92)	6.03 (1.03)	0.921	0.157
	Post	6.63 (0.99)	6.63 (0.81)	6.07 (1.28)	0.107	
	TI^c^	**0.006**	**0.002**	0.902		
	Cohen'd	**0.545**	**0.628**	0.022		
Protrusion	Pre	2.33 (0.54)	2.37 (0.55)	2.33 (0.54)	0.965	**0.049**
	Post	2.93 (0.64)	2.83 (0.59)	2.50 (0.51)	**0.010**	
	TI^c^	**<0.001**	**<0.001**	0.258		
	Cohen'd	**1.204**	**0.919**	0.210		
Sleep quality	Pre	9.83 (3.65)	10.60 (2.19)	8.40 (2.59)	**0.004**	**0.035**
	Post	8.97 (2.89)	10.17 (2.03)	8.40 (2.38)	**0.010**	
	TI^c^	**0.008**	**0.025**	>0.999		
	Cohen'd	**0.517**	**0.430**	0.000		

F: two-way analysis of variance in repeated measures; DN: dry needling; KT: kinesio taping; SD: standard deviation; Post: endline; Pre: baseline; TI: test statistics. Note: p<0.05.

a Comparison between groups.

b Within-group comparison.

c Comparison of baseline and final score differences between groups. Bold values indicate statistically significant differences (p<0.05).

Mouth opening was initially similar (p=0.077), but differed significantly post-treatment (p=0.012). Only the DN group improved significantly (p<0.001); the KT and control groups showed no change (p=0.919; p=0.807). Change scores showed a significant intervention effect (p<0.001) (Table 2).

Maximum mouth opening followed a similar pattern: baseline similarity (p=0.076), significant post-treatment difference (p<0.001), and improvement only in the DN group (p<0.001) (Table 2).

Right and left lateral jaw movements were comparable at baseline (p>0.9). Both DN and KT groups improved significantly (p<0.01), but between-group differences were not significant (right: p=0.088; left: p=0.902) (Table 2).

Protrusion was similar at baseline (p=0.965), but differed post-intervention (p=0.010). DN and KT groups improved (p<0.001); the control group did not (p=0.258). Between-group differences were significant (p=0.049) (Table 2).

Sleep quality differed at baseline (p=0.004) and remained different post-intervention (p=0.010). DN (p=0.008) and KT (p=0.025) groups improved, while the control group did not (p>0.999). Change scores showed significant group differences (p=0.035) (Table 2).

## DISCUSSION

The findings of this study demonstrate that both DN and KT interventions produced significant improvements in pain, sleep quality, and mandibular function among patients with TMD. Notably, the DN group exhibited greater reductions in both VAS and PSQI scores, suggesting superior efficacy in symptom management.

Consistent with our results, Ozmen et al. reported that KT was effective in reducing pain and enhancing functional outcomes in TMD patients in both the medium and long term^19^. This aligns with the observed improvements in mandibular range of motion in our KT group.

The greater therapeutic effect of DN may be attributed to its mechanisms, including trigger point deactivation, increased local circulation, and reduced muscle spasm^14^. Several studies have also confirmed the efficacy of DN, especially when applied to the masseter and temporalis muscles, in alleviating myofascial pain^20^. In addition, while improvements in lateral jaw movements were observed within the DN and KT groups, between-group differences were not statistically significant. Thus, these changes should be interpreted cautiously and not overstated in terms of functional superiority.

Interestingly, slight increases in depression scores were observed in both intervention groups. This finding suggests that short-term physical treatments may have limited effects on psychological parameters. Recent evidence suggests that TMD-related pain is closely linked to emotional distress, especially in the post-pandemic period^21^. In alignment with this biopsychosocial framework, our study observed that both dry needling and KT interventions led to improvements in sleep quality and depressive symptoms, although between-group differences were not statistically significant. In the literature, depression is considered both a cause and a consequence of TMD, underscoring the importance of long-term psychological evaluation^22^.

The improvement in sleep quality, as reflected in PSQI scores, is likely related to the reduction in TMD-related pain. Previous studies have demonstrated the positive effects of relaxation techniques such as facial yoga on sleep quality^14^.

In addition to statistical significance, several outcome measures demonstrated clinically meaningful effect sizes. For example, dry needling (DN) showed a large effect on pain intensity (Cohen's d=2.84) and maximum mouth opening (Cohen's d=1.56), suggesting strong therapeutic benefits. Kinesiotaping (KT) also exhibited moderate effects on depression (d=0.64) and jaw mobility outcomes, although generally lower than DN. These findings support the clinical utility of DN, particularly for improving pain and functional parameters, even in the absence of significant between-group differences for some variables.

Although the research diagnostic criteria for temporomandibular dysfunctions (RDC/TMD) are widely used in clinical and research settings, their diagnostic agreement with imaging modalities such as magnetic resonance imaging (MRI) remains limited. In a previous study, Galhardo et al. reported a diagnostic discrepancy rate of up to 32% between RDC/TMD assessments and MRI findings, particularly in detecting disc displacement and osteoarthritic changes^23^. These findings highlight the need for caution when relying solely on clinical diagnostic tools and emphasize the importance of integrating objective imaging techniques when appropriate. Our study adopted the RDC/TMD criteria due to their practical applicability in clinical settings; however, we acknowledge that this may limit the precision of TMD subgroup classification in the absence of imaging validation.

This study's strengths include its randomized controlled design, the use of a three-arm comparison, and the implementation of interventions by experienced professionals. This study has several important limitations that should be acknowledged. First, the follow-up period was limited to 6 weeks, which may not be sufficient to evaluate the long-term sustainability of treatment effects—particularly for psychological outcomes such as depression and sleep quality. Second, while the sample size was calculated based on expected differences in pain scores, no separate power analysis was conducted for secondary outcomes such as sleep and depression, increasing the risk of Type II error in these domains. Third, the control group received general conservative recommendations (e.g., dietary advice, superficial heat application) but lacked a placebo-equivalent intervention, such as sham taping or sham needling. This design limits the ability to isolate the specific therapeutic effects of DN and KT from non-specific placebo responses. Fourth, all participants were recruited from a single geographic region in Turkey, which may limit the generalizability of the findings to other populations with differing cultural or healthcare contexts. Fifth, the study did not include mechanistic measurements (e.g., electromyography [EMG], biomarkers) to verify the hypothesized physiological effects of DN, such as trigger point deactivation or improved circulation. Finally, the study did not include direct comparisons with gold-standard therapies for TMD, such as occlusal splints or cognitive behavioral therapy (CBT), nor did it address the cost-effectiveness of the interventions. Future research should include longer follow-up, sham-controlled designs, physiological validation measures, and economic evaluations to better understand the clinical and practical value of DN and KT.

Although DN and KT demonstrated clinical benefits in this study, the interventions were not compared with standard treatments such as occlusal splints or CBT, which limits the scope of comparative effectiveness. Additionally, while DN requires specialized clinical training and sterile equipment, KT is a non-invasive, low-cost intervention that can be administered with minimal resources. Future studies should explore not only clinical efficacy but also economic evaluations and head-to-head comparisons with other conservative and gold-standard therapies.

Overall, our findings support the clinical use of DN and KT as effective conservative interventions in the multidisciplinary management of TMD. These techniques, when integrated into individualized treatment protocols, may enhance symptom relief and patient satisfaction. Further research with larger sample sizes and extended follow-up durations is needed to assess the long-term sustainability, mechanisms of action, and cost-effectiveness of these interventions.

## CONCLUSION

Both DN and KT were effective in reducing pain, enhancing jaw mobility, and improving sleep in individuals with TMD. DN showed slightly greater clinical benefits in pain relief and function; however, these findings should be interpreted with caution, as not all between-group comparisons reached statistical significance.

Although improvements in depression and lateral movements were observed within intervention groups, these effects did not differ significantly between groups and therefore should not be interpreted as evidence of superiority.

The slight changes in depression scores underline the importance of addressing psychosocial factors through holistic care. These results support the role of physiotherapy-based conservative methods in multidisciplinary TMD management.

Incorporating DN and KT into personalized treatment plans may enhance outcomes and patient satisfaction. Further research with larger samples and longer follow-up is needed to assess long-term efficacy, mechanisms, and cost-effectiveness.

## ETHICAL STANDARD

Ethical approval was obtained from the Karamanoglu Mehmetbey University's Clinical Research Ethics Committee on [15/01/2025], under the decision number [15-2025/09].

## Data Availability

The datasets generated and/or analyzed during the current study are available from the corresponding author upon reasonable request.

## References

[1] Murphy MK, MacBarb RF, Wong ME, Athanasiou KA (2013). Temporomandibular disorders: a review of etiology, clinical management, and tissue engineering strategies. Int J Oral Maxillofac Implants.

[2] Palmer J, Durham J (2021). Temporomandibular disorders. BJA Educ.

[3] Valesan LF, Da-Cas CD, Réus JC, Denardin ACS, Garanhani RR, Bonotto D (2021). Prevalence of temporomandibular joint disorders: a systematic review and meta-analysis. Clin Oral Investig.

[4] Pantoja LLQ, Toledo IP, Pupo YM, Porporatti AL, Luca Canto G, Zwir LF (2019). Prevalence of degenerative joint disease of the temporomandibular joint: a systematic review. Clin Oral Investig.

[5] Anastassaki Köhler A, Hugoson A, Magnusson T (2012). Prevalence of symptoms indicative of temporomandibular disorders in adults: cross-sectional epidemiological investigations covering two decades. Acta Odontol Scand.

[6] Bueno CH, Pereira DD, Pattussi MP, Grossi PK, Grossi ML (2018). Gender differences in temporomandibular disorders in adult populational studies: a systematic review and meta-analysis. J Oral Rehabil.

[7] Gil-Martínez A, Paris-Alemany A, López-de-Uralde-Villanueva I, Touche R (2018). Management of pain in patients with temporomandibular disorder (TMD): challenges and solutions. J Pain Res.

[8] Chan NHY, Ip CK, Li DTS, Leung YY (2022). Diagnosis and treatment of myogenous temporomandibular disorders: a clinical update. Diagnostics (Basel).

[9] Khan AA, Srivastava A, Passi D, Devi M, Chandra L, Atri M (2018). Management of myofascial pain dysfunction syndrome with meditation and yoga: healing through natural therapy. Natl J Maxillofac Surg.

[10] Kase K, Wallis J, Kase T (2003). Clinical therapeutic application of the kinesio taping method.

[11] Raeissadat SA, Rayegani SM, Sadeghi F, Rahimi-Dehgolan S (2018). Comparison of ozone and lidocaine injection efficacy vs dry needling in myofascial pain syndrome patients. J Pain Res.

[12] Dunning J, Butts R, Mourad F, Young I, Flannagan S, Perreault T (2014). Dry needling: a literature review with implications for clinical practice guidelines. Phys Ther Rev.

[13] Dworkin SF, LeResche L (1992). Research diagnostic criteria for temporomandibular disorders: review, criteria, examinations and specifications, critique. J Craniomandib Disord.

[14] Ozmen EE, Unuvar BS (2024). The effects of dry needling and face yoga on pain, depression, function, and sleep quality in patients with temporomandibular dysfunction. Explore (NY).

[15] Beck AT, Steer RA, Carbin MG (1988). Psychometric properties of the Beck Depression Inventory: twenty-five years of evaluation. Clin Psychol Rev.

[16] Buysse DJ, Reynolds CF, Monk TH, Berman SR, Kupfer DJ (1989). The Pittsburgh Sleep Quality Index: a new instrument for psychiatric practice and research. Psychiatry Res.

[17] Venancio RA, Alencar FG, Zamperini C (2009). Botulinum toxin, lidocaine, and dry-needling injections in patients with myofascial pain and headaches. Cranio.

[18] Hou CR, Tsai LC, Cheng KF, Chung KC, Hong CZ (2002). Immediate effects of various physical therapeutic modalities on cervical myofascial pain and trigger-point sensitivity. Arch Phys Med Rehabil.

[19] Ozmen EE, Durmus E, Unuvar BS, Kalayci A (2022). Mid- and long-term effect of kinesio taping on temporomandibular joint dysfunction: a randomised-controlled trial. Acibadem Univ Health Sci J.

[20] Kietrys DM, Palombaro KM, Azzaretto E, Hubler R, Schaller B, Schlussel JM (2013). Effectiveness of dry needling for upper-quarter myofascial pain: a systematic review and meta-analysis. J Orthop Sports Phys Ther.

[21] Galhardo APM, Andrade PR, Andrade LP, Cury MAA, Mukai MK, Baracat EC (2024). Cross-sectional study of self-reported pain related to temporomandibular disorders and emotional state of medical school faculty and students: post-COVID-19 pandemic. PLoS One.

[22] Manfredini D, Guarda-Nardini L, Winocur E, Piccotti F, Ahlberg J, Lobbezoo F (2011). Research diagnostic criteria for temporomandibular disorders: a systematic review of axis I epidemiologic findings. Oral Surg Oral Med Oral Pathol Oral Radiol Endod.

[23] Galhardo AP, Costa Leite C, Gebrim EM, Gomes RL, Mukai MK, Yamaguchi CA (2013). The correlation of research diagnostic criteria for temporomandibular disorders and magnetic resonance imaging: a study of diagnostic accuracy. Oral Surg Oral Med Oral Pathol Oral Radiol.

